# Gone With the Wind: Exploring a Vanished Rock Dove, *Columba livia*, Hybrid Zone in the Sahara Desert

**DOI:** 10.1002/ece3.72061

**Published:** 2025-08-27

**Authors:** Germán Hernández‐Alonso, Hein van Grouw, Motahare F. Farahani, Torsten Günther

**Affiliations:** ^1^ Human Evolution, Department of Organismal Biology Uppsala University Uppsala Sweden; ^2^ Bird Group, Department of Life Sciences Natural History Museum Tring UK; ^3^ SciLifeLab Ancient DNA Unit, Department of Archaeology and Classical Studies Stockholm University Stockholm Sweden

**Keywords:** hybrid zone, population genomics, refugium theory, speciation *Columba livia*

## Abstract

Rock doves ( *Columba livia* ) are the wild ancestor of domestic and feral pigeons and had a wide distribution across Eurasia and the northern part of Africa. West African rock doves have been identified as genetically distinct from all other populations, possibly representing a distinct species. This divergence is hypothesized to have arisen through cycles of allopatry during the dry and wet Sahara periods. Based on the Refugia Theory and observed admixture patterns, it was proposed that a hybrid zone existed in the Sahara during its last green period, playing a critical role in the speciation of West African rock doves. This project aims to test the existence and location of this vanished hybrid zone by analyzing whole‐genome sequences from six historical rock doves from previously unsampled populations in the Central Sahara and West Africa, along with published genomic data. By exploring population structure, genetic diversity, and admixture patterns, our results confirm the existence of the hybrid zone, likely located around the mountainous regions of northwest Africa. To explain the observed genetic differentiation of West African rock doves, we propose a four‐step scenario involving speciation by reinforcement. Finally, we support a species‐level taxonomic arrangement to designate the West African rock dove as *C. gymnocycla*.

## Graphical abstract

In this study, we tested the hypothesis of the existence of a hybrid zone between two differentiated rock dove lineages, which may contribute significantly to their divergence. By analyzing newly generated genomic data from previously unsampled African rock dove populations, along with publicly available rock dove genomes, we provide evidence for the existence of a hybrid zone in West Africa, likely established during the last Holocene Green Sahara Period.

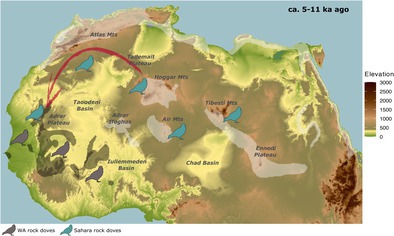

## 1 Introduction

The rock dove, *Columba livia* , better known for being the parental species of the domestic and feral pigeons, has a historical native range widely spread across the middle and lower latitudes of the West Palearctic and the Atlantic Islands, mainly associated with nesting sites on rocky areas like caves or cliffs (Cramp 1985). Currently, nine subspecies are commonly recognized; however, a recent genomic study of the species has suggested several taxonomic rearrangements (Hernández‐Alonso et al. 2023). This study identified a divergent population in the west of Africa that could represent a distinct species (*C. gymnocycla*), while finding low genetic differentiation among the remaining rock dove populations. Additionally, the likely feral origin of one recognized subspecies and two other inconclusive subspecies was detected. These include *dakhlae* from the Dakhla Oasis, Egypt, *atlantis* from Azores, Madeira, and Cape Verde, *canariensis* from the Canary Islands.

The West African (WA) rock dove, currently isolated from other populations by the Sahara Desert, is morphologically distinguished by its dark colouration and an extended, bright scarlet orbital ring (Gibbs et al. 2001). In addition to its high genetic differentiation, a significant genetic distinction between the WA rock doves and other populations is the absence of genetic introgression from *C. rupestris* , the hill pigeon, which is distributed in Central and East Asia. In contrast, all other rock dove genomes analysed show strong signals of admixture with the hill pigeon. These admixture patterns, observed in geographically distant populations, suggest an ancient admixture event between the hill pigeon and the common ancestor of the rock doves, after the split from the WA rock doves (Hernández‐Alonso et al. 2023).

Based on the Refugium Theory, it has been suggested that the WA rock doves could represent an ancestral lineage that diverged through allopatric cycles driven by the wet‐dry periods of the Sahara during the Pleistocene epoch (Hernández‐Alonso et al. 2023). This evolutionary scenario suggests that during the Last Glacial Maximum (LGM) (ca. 18,000 years ago), WA rock doves likely survived in more stable regions south of the Sahel in West Africa, while other rock dove populations were restricted to a hypothetical refugium in Central Asia, where admixture with the hill pigeon occurred. After the LGM, climatic conditions became more favorable, allowing the two differentiated lineages to expand, particularly the Central Asian lineage. During the Holocene Green Sahara Period (ca. 5000–11,700 years ago) (Skonieczny et al. 2015), WA and Central Asian rock doves may have come into secondary contact in the Sahara region, forming a hybrid zone. Notably, it has been suggested that hybrid zones may retard or prevent the mixing of differentiated lineages, preserving their genetic integrity. Climate changes can reduce the lineages distribution once again, contributing to the speciation processes (Futuyma and Shapiro 1995; Hewitt 1996), as could happen in the case of the WA rock doves. The underlying mechanisms associated with this phenomenon are linked to reduced fitness in hybrids, either due to environmental factors or genetic incompatibilities (Futuyma and Shapiro 1995). Finally, the current dry Sahara period vanished the hybrid zone, resulting in the modern rock doves' distribution.

To better understand the evolutionary history of rock doves, this study investigates the existence of a hybrid zone between the WA rock doves and the Central Asian lineage in the Sahara. Additionally, we aim to gather evidence supporting the proposed species‐level reclassification of WA rock doves as *C. gymnocycla*. By generating whole‐genome data from previously unsampled Sahara rock doves and WA populations from Senegal, we expect to detect differentiated admixture proportions between the WA and Sahara rock doves along their geographic locations, revealing distribution range shifts, gene flow patterns, and the approximate location of the hybrid zone.

## 2 Materials and Methods

### 2.1 Dataset

**FIGURE 1:**
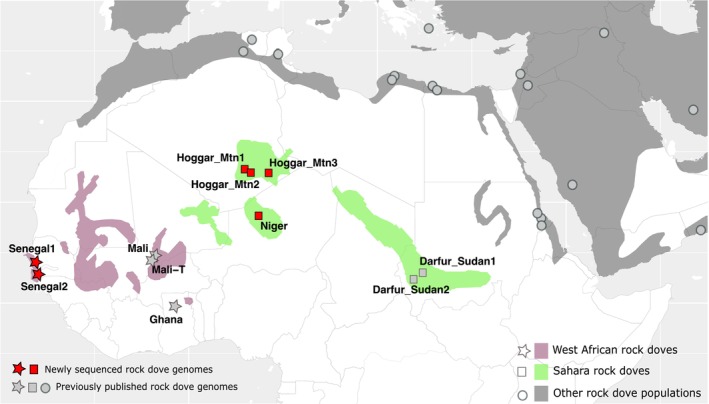
Geographic locations of rock dove samples from Africa and adjacent regions. The map highlights the estimated geographic distribution ranges of various rock dove populations according to Urban et al. (1986): West African and Saharan rock doves, as well as other populations. Previously published genome sample locations are marked in grey, while the locations of newly sequenced samples are shown in red.

Six dry toepads were obtained from historical rock dove specimens housed at the Natural History Museum in Tring, UK, dating from the 19th and early 20th centuries (Table S1). These specimens represent previously unsampled WA rock dove populations from Senegal, including a paratype specimen (Senegal‐P), and Sahara rock dove populations from the Hoggar Mountains (Algeria) and Aïr Mountains (Niger) (Figure 1).

For comparison, we included the genome of 28 reference rock dove samples from Africa and other regions within the species' historical distribution (Hernández‐Alonso et al. 2023). To further capture the diversity of domestic pigeon populations, 12 genomes from various geographic and traditional breed groups were included (Shapiro et al. 2013). Lastly, five Columbidae outgroup species were added to the dataset: one hill pigeon ( *C. rupestris* ) (Shapiro et al. 2013), one speckled pigeon ( *C. guinea* ), one common wood pigeon ( *C. palumbus* ), one cinnamon dove ( *C. larvata* ) (Vickrey et al. 2018), and one band‐tailed pigeon ( *Patagioenas fasciata* ) (Murray et al. 2017) (Table S2).

### 2.2 Laboratory Procedures

DNA extraction and library preparation for sequencing were conducted by the SciLifeLab Ancient DNA unit at the dedicated ancient DNA laboratory within the Centre for Palaeogenetics (CPG), Stockholm, Sweden.

DNA extraction was performed on six toe pads from rock doves using a modified protocol based on Sinding et al. (2015). The digestion buffer comprised 10 μL of 1 M Tris–HCl (pH 8), 2 μL of 5 M NaCl, 5 μL of 1 M CaCl2, 5 μL of 0.5 M EDTA (pH 8), 200 μL of 10% SDS, 10 μL of DTT (Cleland's reagent), 100 μL of 10% Proteinase K solution (e.g., > 600 mAU/mL, Qiagen), and 678 μL of lab‐grade H2O. Samples were incubated overnight at 56°C. To ensure quality control, an extraction and library blank were included as negative controls throughout the process.

For the second stage of extraction, protocols from Yang et al. (1998) and Svensson et al. (2021) were followed, using Vivaspin columns (Sartorius). Washing steps utilized Qiagen PB and PE buffers, and the final extracts were eluted in 110 μL of EB.

Library preparation for next‐generation sequencing was performed to create double‐stranded, blunt‐end libraries using a previously published protocol (Meyer and Kircher 2010). Enzymatic reactions were cleaned using the MinElute PCR purification kit instead of SPRI beads. Rigorous measures were taken to prevent cross‐contamination, including the use of negative controls (extraction blanks) and PCR blanks at every step of library preparation and amplification. Real‐time PCR (qPCR) was employed to optimize the number of PCR cycles for each library.

Dual‐indexing PCR amplification was conducted in duplicates using a 50 μL reaction mix, which included 6 μL of the DNA library, 5 U of AmpliTaq Gold DNA polymerase (Thermo Fisher Scientific), 1× GeneAmp Gold buffer (Thermo Fisher Scientific), 2.5 mM MgCl2, 250 μM of each dNTP, and 200 nM each of the P7 and P5 indexing primers (Kircher et al. 2012). Libraries were quantified using the 4150 TapeStation System (Agilent). Sequencing was carried out using a 10B lane with 2 × 150 bp paired‐end reads on the Illumina NovaSeq X platform at the SciLifeLab sequencing facilities in Stockholm, Sweden.

### 2.3 Data Processing

To remove sequencing adaptors and the commonly observed Illumina NextSeqX high‐quality polyG tails (Chen et al. 2018) from the newly generated sequence reads, we used Cutadapt v.4.4 (Martin 2011) with the parameters: *‐‐quality‐base 33*, ‐‐nextseq *‐trim = 15*, ‐‐overlap *3*, ‐e *0.2*, ‐‐trim *‐n*, and *‐‐minimum‐length 15:15*. Then, FLASH v.2.11 (Magoč and Salzberg 2011) was implemented for merging paired‐end reads using the parameters *‐‐min‐overlap 11*, ‐‐max *‐overlap 150*. After this step, all sequence reads were mapped to the *C. livia* reference genome (Cliv_2.1) (Holt et al. 2018) using PALEOMIX v.1.3.6 BAM pipeline (Schubert et al. 2014). The pipeline includes (1) the sequencing adapters trimming and discard of reads shorter than 25 bp using AdapterRemoval v.2.2.0 (Schubert et al. 2016); (2) alignment of sequence reads to the reference genome as implemented in BWA v.0.7.17 backtrack algorithm (Li and Durbin 2009) disabling the seed parameter and discarding unmapped reads; (3) PCR duplicates removal using Picard's MarkDuplicates (https://github.com/broadinstitute/picard); (4) local realignment around indels using the module GATK v.3.8.3 IndelsRealigner (McKenna et al. 2010); (5) estimation of nucleotide misincorporation patterns and DNA fragmentation in the newly generated historical rock dove sequence reads using mapDamage v.2.0.9 (Jónsson et al. 2013) (Table S1).

### 2.4 Sex Determination

The biological sex of the six rock dove specimens was determined by mapping sequence reads to the chromosome‐level *C. livia* reference genome (colLiv2) (Damas et al. 2017) using the same PALEOMIX BAM pipeline described above. If the depth of coverage for the Z sex chromosome was similar to or higher than the mean coverage of the autosomal chromosomes, the specimen was identified as male. If the Z chromosome depth of coverage was approximately half the mean autosomal coverage, the specimen was identified as female (see Table S1).

### 2.5 SNP Calling

Given the low to medium depth of coverage of the rock dove nuclear genomes, SNP calling was performed using a pseudo‐haploid approach. To achieve this, ANGSD v.0.933 (Korneliussen et al. 2014) with the function *‐dohaplocall 1* was used to randomly sample one read per site for all samples in the dataset. The sites were selected according to the following parameters: *‐doCounts 1*, *‐minMinor 2*, *‐maxMis 10*, *‐C 50*, *‐baq 1*, *‐minMapQ 30*, *‐minQ 20*, *‐uniqueOnly 1*, *‐remove_bads 1*, *‐only_proper_pairs 1*, *‐skipTriallelic 1*, *‐doMajorMinor 1*, and *‐GL 2*. Additionally, the parameter *‐rmTrans 1* was used to remove transitions commonly found in historical samples due to ancient DNA damage. SNP calling was limited to the first 128 scaffolds with a minimum length of 1 Mb. Subsequently, sites with a minor allele frequency below 0.01 were removed using Plink v.1.90 (Chang et al. 2015), resulting in a final dataset of 3,763,663 transversion sites (11.46% ± 0.019 SE missingness per individual on average).

### 2.6 Dimensionality Reduction Analyses

The population structure in the dataset was explored using multidimensional scaling (MDS) and principal component analyses (PCA). For the MDS analysis, we estimated pairwise identity‐by‐state (IBS) genetic distances from the SNP dataset using Plink v.1.9, excluding all outgroups from the dataset. A *Stress‐1* value was calculated for the two‐dimensional MDS using the R library SMACOF v.2.1‐7 (De Leeuw and de Leeuw and Mair 2009). A *Stress‐1* value of 0.17 was obtained, which falls below the commonly accepted threshold of 0.20 (Borg and Groenen 2005).

For generating the PCA, genotype likelihoods were estimated using ANGSD v.0.933 with parameters *‐GL 2*, ‐doGlf *2*, ‐doMajorMinor *1*, ‐doMaf *2*, *‐doCounts 1*, *‐SNP_pval 1e‐6*, ‐rmTrans *1*, ‐minQ *20*, ‐minmapq *30*, *‐setMinDepth 3*, *‐uniqueOnly 1*, ‐remove_bads *1*, ‐only_proper_pairs *1*, *‐C 50*, ‐baq *1*, ‐skipTriallelic *1*, and *‐minMaf 0.00001*. Then, PCAngsd v.1.11 (Meisner and Albrechtsen 2018) was used to generate a covariance matrix. Finally, the R function *eigen* was used to calculate eigenvalues.

### 2.7 Individual Ancestry Component Estimation

**FIGURE 2:**
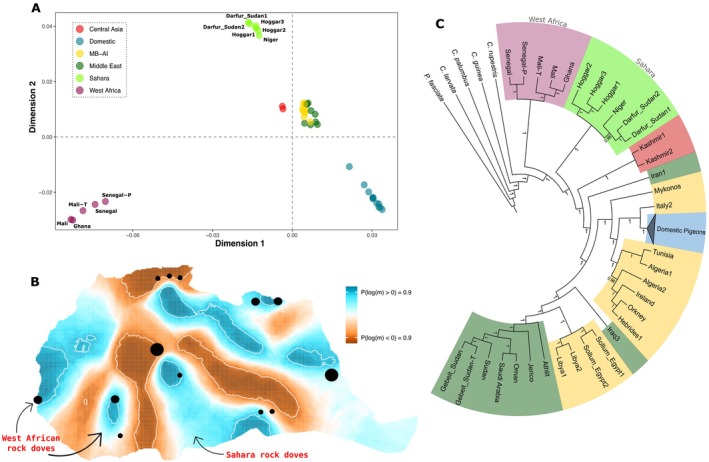
Population structure and phylogenetic affinities of rock doves. (A) MDS plot displaying the genetic relationships among all rock doves and domestic pigeons in the dataset. MB‐AI stands for Mediterranean Basin and Atlantic Islands. (B) EEMS plot focused on African rock doves, illustrating the posterior log mean migration rates. The white polygons represent 95% confidence intervals. White areas indicate average migration rates consistent with a model of isolation by distance, while blue regions reflect higher‐than‐average migration rates, and orange regions indicate lower‐than‐average migration rates. Sample locations are shown as circles, with circle size representing the number of samples per site (1, 2, or 3). (C) Neighbour‐joining phylogenetic tree constructed from genomic pairwise distances. The domestic pigeon clade is collapsed for clarity. Bootstrap support values are shown for each internal node. Colours correspond to the main geographic regions as defined in the MDS plot.

To investigate shared ancestry components in the WA and Sahara rock doves, we implemented ADMIXTURE v.1.3.0 (Alexander et al. 2009) assuming 2 to 5 ancestry components (*K* = {2…5}) based on the population structure observed in the phylogenetic tree, MDS, and PCA (Figure 2A,B; Figure S1). Ten replicates were performed, and for each K, the replicate with the best likelihood value was chosen. The SNP dataset (excluding outgroups) was used as input. To visualize the results, we used the R library Pophelper v.2.3.1 (Francis 2017).

### 2.8 Estimated Effective Migration Surface (EEMS)

EEMS (Petkova et al. 2016) was used to explore the population structure and gene flow patterns of the rock doves from Africa in our dataset in relation to their geographic distribution. This analysis allows for the detection of potential barriers or corridors between populations, based on whether estimated migration rates are lower or higher than the average migration rates under a model of isolation by distance. To run EEMS, sites with missing data were removed from the SNP dataset (*‐‐geno 0.9999*), obtaining a final dataset of 1,795,203 SNPs. Then, a dissimilarity matrix was estimated using EEMS' function *bed2diffs*. The following parameters were used to generate the analysis: nInd = 21, nSites = 1,795,203, nDemes = 200, diploid = false, numMC‐MCIter = 2,000,000, numBurnIter = 1,000,000, numThinIter = 9999. Results were visualized using the R library reemsplots2 v.0.1.0 (https://github.com/dipetkov/reemsplots2).

### 2.9 Neighbour‐Joining Tree

A phylogenetic tree was estimated using the neighbour‐joining (NJ) algorithm (Saitou and Nei 1987) and 100 pairwise‐distance matrices calculated in Plink v.1.90 by randomly sampling 1 million sites from the SNP dataset. Each NJ tree was estimated using the *nj* function as implemented in the R library ape v.5.7‐1 (Paradis and Schliep 2019); then, all trees were summarized using Astral‐III (Zhang et al. 2018). The final tree was visualized using the Interactive Tree Of Life (iTOL) v.4 online tool (Letunic and Bork 2019).

### 2.10 Gene Flow Analyses

To explore admixture patterns between rock dove populations and test distinct hypotheses about their evolutionary history, we used the *D*‐statistic as implemented in ADMIXTOOLS v.7.0.1 (Patterson et al. 2012). If a test in the form *D* (Outgroup, A; B, C) results in a *D* < 0, it indicates possible gene flow between A and B; if *D* > 0, it indicates possible gene flow between A and C. Deviations from 0 were considered statistically significant when the Z‐score was under −3 or above 3. The test's significance was calculated using a weighted block jackknife procedure over 1 Mb blocks. In all the tests, domestic pigeons were grouped into a single population. Four hypotheses were tested:

1. To prove that all the Sahara rock doves present admixture signals with *C. rupestris* when compared to the WA rock doves, indicating they belong to the rock dove's lineage that originated in Central Asia, we performed the test in the form *D* ( *C. palumbus* , *C. rupestris* ; WA, X). All WA rock doves were grouped as a single population; X represents all rock doves and domestic pigeons in the dataset.
2. To explore the possible admixture between WA and Sahara rock doves, a test was conducted following the obtained NJ tree topology: *D* ( *C. palumbus* , WA; Sahara, X) where X is rock doves. For this test, each genome was tested individually.
3. To determine the directionality of the admixture between Sahara and WA rock doves, which could suggest the location and extent of the hybrid zone in the Sahara Desert, a first test was performed in the form *D* ( *C. palumbus* , WA; Sahara1, Sahara2). All WA rock doves were grouped, and Sahara1/2 represent different Sahara rock dove genomes. Similarly, another test was performed in the form *D* ( *C. palumbus* , Sahara; WA1, WA2), where all Sahara rock doves were grouped in a single population, and WA1/2 represent different combinations of WA rock dove genomes.
4. Finally, to test possible admixture between WA rock doves and domestic pigeons, two sets of tests were performed in the form *D* ( *C. palumbus* , WA; domestic pigeons, ECA rock doves) and *D* ( *C. palumbus* , WA; domestic pigeons, Sahara rock doves), where ECA represents the grouped rock doves from Eurasia and coastal Africa (Mediterranean and Red Sea).

Additionally, admixture graphs were estimated to detect migration events among the distinct rock dove populations. For this purpose, we used the software OrientAGraph v.1.0 (Molloy et al. 2021) which works as an extension of TreeMix (Pickrell and Pritchard 2012), improving the accuracy of the estimated tree topologies and producing graphs with higher likelihood scores by implementing a maximum likelihood network orientation algorithm. For this analysis, the outgroups were removed from the SNP dataset, except for *C. palumbus* , and sites with missing data were removed, resulting in 98,373 SNPs. Samples from the same location were grouped into single populations. OrientAGraph estimated 0–4 migration events (*‐m*) adding the parameter *‐mlno* to run maximum likelihood network orientation after each migration edge. Ten replicas were performed, and the results with the best likelihood values were chosen. The results were plotted on R using TreeMix script plotting_funcs.R.

### 2.11 Genetic Structure and Diversity Assessment

The pairwise differentiation index (*F*_ST_) was estimated to assess genetic differentiation among distinct rock dove populations using the SNP dataset and the Plink *‐fst* function. Populations were defined by selecting two rock dove genomes per geographic region: Senegal (Senegal‐P, Senegal), Mali (Mali‐T, Mali), Hoggar Mountains (Hoggar1, Hoggar3), Darfur (Darfur_Sudan1, Darfur_Sudan2), Gebeit (Gebeit_Sudan, Gebeit_Sudan‐T), Egypt (Sollum_Egypt1, Sollum_Egypt2), Algeria (Algeria1, Algeria2), Libya (Libya1, Libya2), Levant (Athlit, Jerico), Scotland (Hebrides1, Orkney), and Kashmir (Kashmir1, Kashmir2).

To calculate individual inbreeding coefficients of the rock doves in our dataset, we used ngsRelate v.2.0 (Hanghøj et al. 2019), which infers inbreeding coefficients (*F*) from low‐coverage genomes by using genotype likelihoods. Genotype likelihoods were estimated with ANGSD v.0.933 as described previously, except with the parameters *‐doGlf 3* and *‐doMaf 1*, as recommended in the ngsRelate manual.

To avoid biases in heterozygosity estimation per sample due to coverage depth differences among rock dove samples, read alignments were downsampled to retain 10 million randomly selected mapped reads using Picard's *DownsampleSam* function. After downsampling, genotype likelihoods were calculated for each sample at the outgroup segregating sites using ANGSD v.0.933 with the parameters *‐dosaf 1*, ‐gl *2*, *‐C 50*, ‐minQ *20*, ‐minmapq *30*, *‐doCounts 1*, and *‐setMinDepth 3*. Finally, the site frequency spectrum (SFS) was estimated using ANGSD realSFS and used as an approximation for individual heterozygosity.

To identify signals of demographic expansion or contraction, Tajima's D was estimated. Three rock dove populations were defined: West African (Mali, Mali‐T, Senegal, Senegal‐P, Ghana), Sahara (Hoggar1, Hoggar2, Hoggar3, Niger, Darfur_Sudan1, Darfur_Sudan2), and North African (Sollum_Egypt1, Sollum_Egypt2, Libya1, Libya2, Algeria1, Algeria2, Tunes). Site allele frequency likelihoods were calculated for each population in ANGSD with the parameters *‐doSaf 1* and ‐*GL 1*. Due to the lack of an ancestral state genome (*‐anc*), we used the *C. livia* reference genome. Then, the folded spectrum (*‐fold 1*) was calculated using ANGSD realSFS, as recommended when the ancestral state is unknown (Korneliussen et al. 2013). Subsequently, theta values were calculated for each site using the realSFS function *saf2theta*, and finally, Tajima's D was estimated in 50,000 bp windows with 10,000 bp steps by using the command *thetaStat do_stat ‐win 50,000 ‐step 10,000*. Normality of the data was assessed using the Anderson‐Darling test, as implemented in the R library nortest v.1.0‐4 (Gross and Ligges 2015). The test indicated a non‐normal distribution for each set of results (*A* = 481.73; *p*‐value < 2.2e−16). Subsequently, a Kruskal–Wallis test was performed in R (*kruskal.test*) to evaluate statistical differences among the three populations. The test revealed a strong difference among the three populations (chi‐squared = 181,256; *p*‐value < 2.2e−16). Finally, post hoc pairwise comparisons were conducted using the Wilcoxon rank‐sum test in R (*pairwise.wilcox.test*) with Benjamini‐Hochberg (BH) correction to identify which population pairs differed significantly. All pairwise comparisons were highly significant (*p*‐value < 2e−16), indicating that the distribution of Tajima's *D* values differs between every pair of populations.

## 3 Results

From the DNA extracted from the six toe pads, we sequenced between 135,310,794 and 208,948,341 Illumina pair reads per sample. After mapping to the *C. livia* reference genome, we obtained an average depth of coverage of 2.56X ± 0.33 SE across the samples (1.42–3.41X). Based on the coverage of the Z chromosome, three samples are considered male and three are female. From our 51 Columbidae genomes dataset, we generated a SNP panel containing 3,763,663 transversion sites.

### 3.1 Rock Doves' Divergence and Isolation Across Northern Africa

The population structure shown in the MDS plot recovered four main clusters: domestic pigeons, Eurasian and coastal African (ECA) rock doves, Sahara rock doves, and WA rock doves. The WA rock dove genomes appear highly divergent, separated along both dimensions, while the Sahara rock doves are separated from ECA rock doves along dimension 2 (Figure 2A). This is further supported by the results of other exploratory analyses. For instance, the PCA estimated with genotype likelihoods shows similar patterns of multidimensional segregation: WA rock doves are separated along principal component (PC) 2, while Sahara rock doves are slightly separated from other rock doves along PC1 (Figure S1). Similarly, model‐based ancestry assignment analyses (Figure S2), performed to estimate individual ancestry composition, define an independent WA rock dove cluster when the analyses are run with 3 to 5 ancestry components, without signals of admixture with other ancestry components. Sahara rock doves cluster with other rock doves when 3 ancestry components are estimated, while they separate into a new independent cluster, without signals of admixture, when the analyses are performed for 4 and 5 ancestry components.

The exploration of geographic gene flow patterns of the rock doves from Africa with EEMS revealed potential barriers among the WA, Sahara, and other African rock doves, most likely associated with the Sahara Desert. WA rock doves from Senegal seem isolated from the populations in Mali and Ghana. On the other hand, Sahara rock doves show a higher migration rate than the average among themselves, but lower in relation to other coastal African populations. Finally, high migration rates were estimated among the remaining coastal African rock doves connecting the Red Sea and the Mediterranean region (Figure 2B).

In agreement with the EEMS results, pairwise F_ST_ estimates between WA rock doves and any other rock dove populations indicate very high genetic differentiation (mean *F*_ST_ = 0.347 ± 0.013 SE). Comparisons between the populations from Mali and Senegal yield a moderate *F*_ST_ value (0.15). Moderate to high levels of differentiation (mean *F*_ST_ = 0.162 ± 0.009 SE) were observed between Sahara rock doves and ECA rock dove populations. In contrast, *F*_ST_ values between the ECA rock doves indicated low to moderate genetic differentiation (mean *F*_ST_ = 0.071 ± 0.008 SE) (Figure S3).

This pattern of population differentiation is also visible in the inferred NJ tree, where WA rock doves form a monophyletic clade that represents the earliest diverging lineage within this species complex. Furthermore, the Sahara rock doves also appear monophyletic, forming the sister clade to the ECA rock doves and domestic pigeons. All branches in the tree obtained high bootstrap support values (Figure 2C).

### 3.2 Exploring the Genetic Diversity of Rock Doves

**FIGURE 3:**
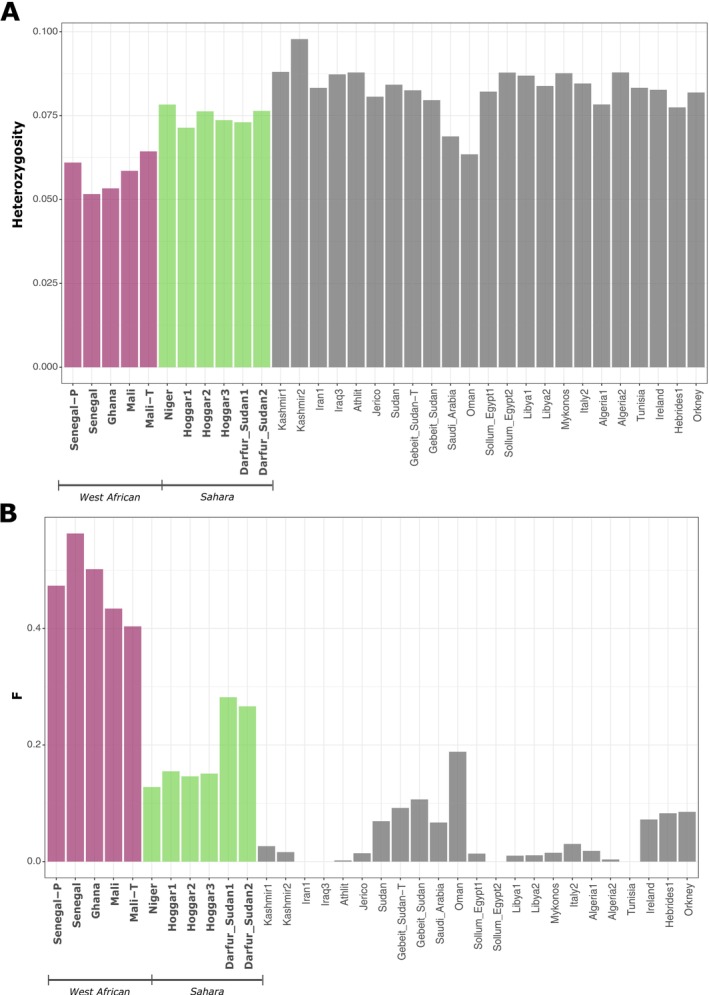
Genetic diversity assessment. (A) Global per‐sample heterozygosity calculated from the SFS. WA rock doves exhibit the lowest heterozygosity values, followed by Saharan rock doves. (B) Individual inbreeding coefficient (*F*) estimated from genotype‐likelihoods. The WA rock doves present the highest *F* values, followed by the Sahara rock doves. Other tested populations exhibit low to moderate *F* values.

The estimated global per‐sample heterozygosity revealed that the WA populations exhibit the lowest heterozygosity values (0.051–0.064; mean = 0.057 ± 0.002 SE), followed by the Sahara rock doves (0.071–0.078; mean = 0.075 ± 0.001 SE). In contrast, the remaining rock dove populations in the dataset showed higher heterozygosity, generally exceeding 0.075, except for the samples from the Arabian Peninsula (Oman = 0.063 and Saudi Arabia = 0.068) (Figure 3A).

Consistent with the heterozygosity results, the estimated inbreeding coefficients indicate that the WA populations have very high *F* values (0.4–0.56; mean = 0.475 ± 0.028 SE) (Figure 3B), while the Sahara rock doves exhibit moderate to high values (0.12–0.28; mean = 0.188 ± 0.028 SE). Most rock dove populations displayed *F* values below 0.1, except for the sample from Oman (*F* = 0.18).

To explore population histories, a per‐window Tajima's *D* neutrality test was applied to three African rock dove populations. The estimated Tajima's *D* values for all populations are negative, apart from some outliers. The per‐population medians for Tajima's *D* values are as follows: African Mediterranean = −1.002, Sahara = −1.118, and WA = −0.557. These results suggest an excess of rare variants, consistent with either population growth or a non‐recent bottleneck (Figure S4).

### 3.3 Admixture Patterns Reveal Gene Flow Directionality Between WA and Sahara Rock Doves

**FIGURE 4:**
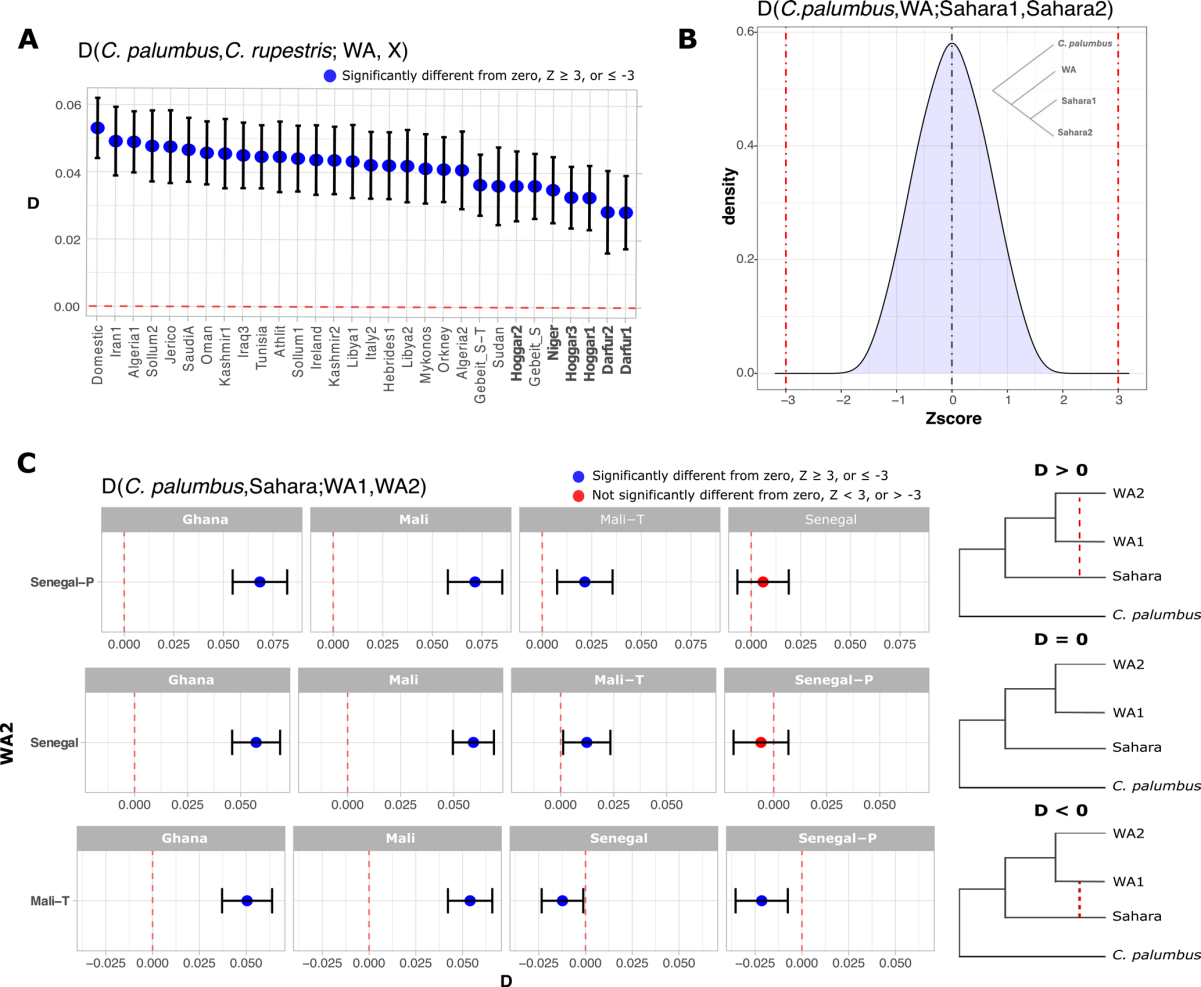
Admixture analysis using *D*‐statistics. (A) D‐statistic test exploring potential admixture signals between Saharan rock doves and *C. rupestris* . (B, C) *D*‐statistic tests exploring admixture between WA and Sahara rock doves. (B) The distribution of 30 Z‐scores is centered around zero, and most values are < |2|, indicating no detectable admixture signals when comparing WA rock doves against distinct Sahara rock doves. (C) When the test configuration is reversed, the resulting *D* values reveal differential admixture signals among WA rock doves when compared to the Sahara rock doves. Notably, Senegal rock doves exhibit the strongest admixture signals compared to other WA samples, followed by the Mali‐T sample.

To investigate whether Sahara rock doves belong to the Central Asia rock dove's lineage that admixed with *C. rupestris* , we used *D*‐statistics in the form *D* ( *C. palumbus* , *C. rupestris* ; WA, X) where X are the different individual rock doves. All obtained *D* values were positive and statistically significant. These results indicate that Sahara rock doves, like other rock doves and domestic pigeons in the dataset, share more alleles than expected with *C. rupestris* when compared to the WA rock doves. This suggests that Sahara rock doves belong to the Central Asian rock dove lineage (Figure 4A).

Next, we investigated potential admixture between WA and Sahara rock doves using *D*‐statistics in the form *D* ( *C. palumbus* , WA; Sahara, X). The obtained *D* values were generally negative and, in most cases, statistically significant. The results suggest that all tested WA rock doves share more alleles than expected with Sahara rock doves when compared to other rock doves (Figures S5 and S6).

Once the admixture between WA and Sahara rock doves was confirmed, we implemented additional *D*‐statistics to explore the directionality of gene flow and detect differential levels of admixture among populations. The first set of tests in the form *D* ( *C. palumbus* , WA; Sahara1, Sahara2) generated values not statistically different from zero, failing to detect differences in the admixture signals between Saharan individuals (Figure 4B). However, when the test configuration was reversed in the form *D* ( *C. palumbus* , Sahara; WA1, WA2), comparing Sahara rock doves with paired WA rock doves, statistically significant *D* values indicate variation in the admixture strength signals among WA populations. Specifically, the two samples from Senegal exhibited the strongest admixture signals compared to other WA rock doves, followed by the Mali‐*T* sample (Figure 4C).

To confirm that the differential admixture signals observed in WA rock doves are not associated with admixture from feral or domestic pigeons, additional *D*‐statistics tests were performed. When comparing each WA rock dove sample with domestic pigeons and either Sahara or ECA rock doves *D* ( *C. palumbus* , WA; domestic pigeons, ECA) and *D* ( *C. palumbus* , WA; domestic pigeons, Sahara), all resulting *D* values were positive and statistically significant. These results indicate more allele sharing with wild than domestic rock doves, suggesting that the observed admixture signals in the WA rock dove genomes originate from wild rock doves, not domestic pigeons. Furthermore, the *D* values were consistently higher when Sahara rock doves were used in the tests (Figure S7).

Finally, admixture graphs generated with OrientAGraph corroborate admixture between the WA rock doves and the Sahara rock doves. The estimated tree topologies are consistent with the NJ tree structure and the geographic distribution of the populations used in these analyses (Figures S8 and S9). Overall, the WA rock dove clade presents consistent admixture signals with the Sahara rock doves, indicating admixture from the common ancestor of WA populations into the Sahara rock dove lineage.

## 4 Discussion

### 4.1 A Vanished Hybrid Zone in the Western Sahara

The obtained results support the hypothesis that a hybrid zone once existed between the Central Asian and WA rock dove lineages, likely formed after the LGM (ca. 10,000 years ago), when the climate conditions became more favorable. This is consistent with the Refugium Theory (Hewitt 1996; Mayr and O'Hara 1986), which predicts the establishment of hybrid zones following isolation during the glacial periods. Our genomic data confirmed the previously reported admixture between WA and Sahara rock doves from Sudan (Hernández‐Alonso et al. 2023), allowing us to suggest an approximate location of the hybrid zone in the West of Africa.

The sequencing of the new rock dove genomes allowed us to confirm that the Sahara rock doves belong to the Central Asian rock dove lineage that admixed with *C. rupestris*, as shown by the *D*‐statistic results (Figure 4A). On the other hand, WA rock doves form a genetically differentiated monophyletic group without admixture from *C. rupestris* , supporting the independence and high divergence of the two lineages. Also, the results confirmed the admixture between the Sahara and WA rock doves, revealing unidirectional admixture from the Sahara rock doves into the WA rock doves (Figure 4B,C).

**FIGURE 5:**
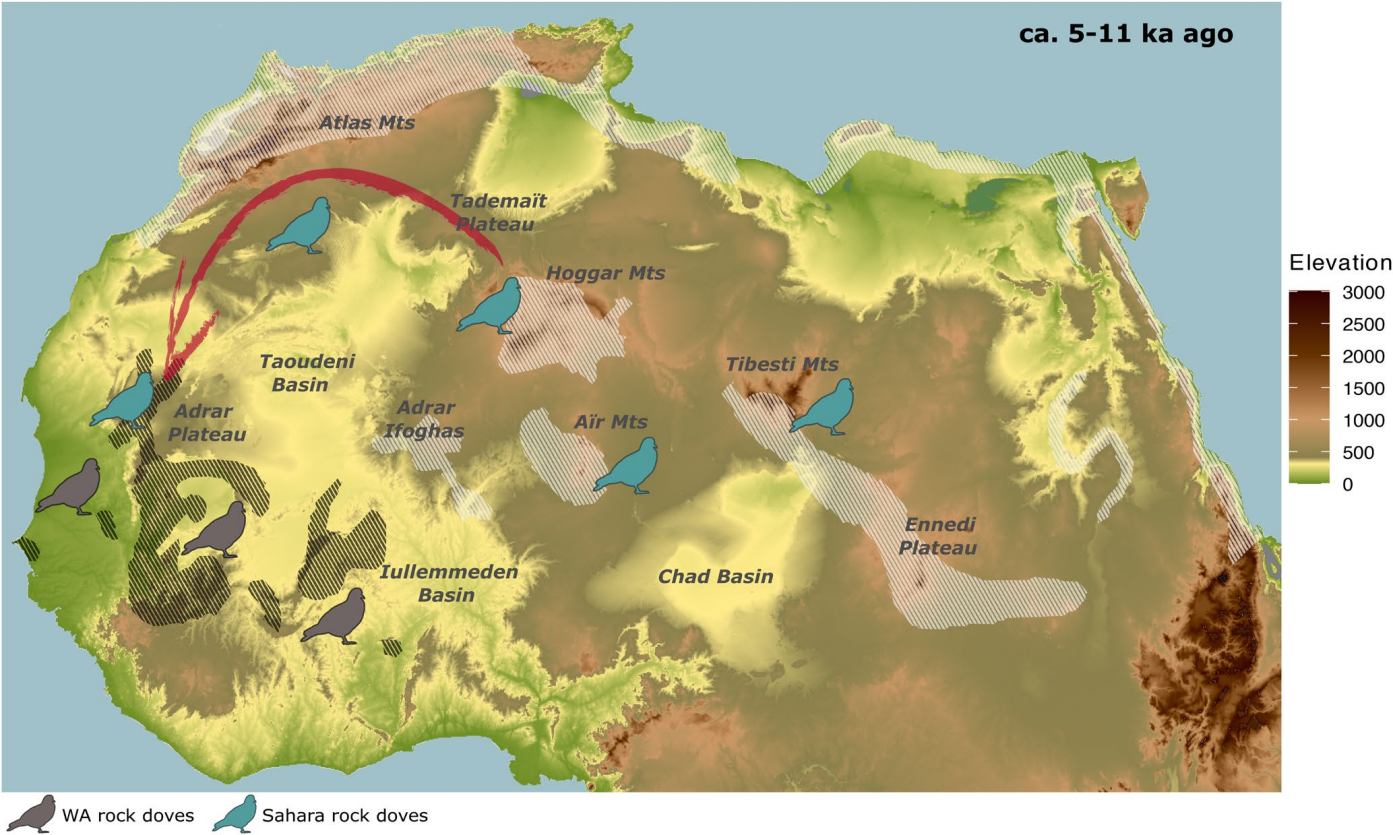
Schematic model of the Sahara rock dove expansion into West Africa during the Holocene Green Sahara Period (ca. 5000 to 11,700 years ago). The map depicts elevations across Northern Africa, including major mountains, plateaus, and basins. Shaded areas represent the modern rock dove's geographic distribution, with black indicating the range of WA rock doves and white representing other African rock dove populations. The model suggests that the expansion of rock doves from the Central Sahara to the west followed rocky landscapes in northwest Africa, which provided suitable habitats for resting and nesting (indicated by the red arrow). This movement likely occurred via the Tademït Plateau and the Atlas Mountains, eventually reaching the Adrar Plateau. Hybridisation with WA rock doves may have taken place around this region.

During the Holocene Green Sahara period (ca. 5000–11,700 years ago), favorable environmental conditions likely enabled rock doves already distributed across the Sahara to keep expanding westward, following rocky areas necessary for nesting and resting. A possible route to the west included the Tademaït Plateau, the Atlas Mountains, and the Adrar Plateau (Figure 5). Conversely, due to unsuitable conditions, their mobility may have been limited across the vast, flat regions of the Taoudeni and Iullemmeden Basins, which were covered by lakes, swamps, and rivers at the time (Skonieczny et al. 2015; Fabre and Petit‐Maire 1988) (Figure 5).

This invasion of the west of Africa established the secondary contact and subsequent hybrid zone likely around the mountainous regions of Mauritania, which the WA rock doves are thought to inhabit (Figure 5). However, there is no clear evidence of WA rock doves' presence in the Mauritanian mountains yet (van Grouw and Hernández‐Alonso 2025). Therefore, the hybrid zone could be alternatively established at the south of the Mauritanian mountains, where the hilly and rocky areas decrease. Later, the current dry period reduced the Sahara rock dove's distribution, vanishing the hybrid zone. The negative Tajima's *D* values may reflect the population recovery after the bottleneck produced by the Sahara aridification (Figure S4).

The differential admixture signals among the WA rock doves, with Senegalese populations showing the strongest signals, can be explained by their proximity to the potential location of the vanished hybrid zone. Additionally, the observed admixture gradient indicates that the most isolated populations, such as those in Ghana, exhibit weaker admixture signals with the Sahara rock doves. These results point out that WA rock dove populations had higher connectivity and wider distribution in the West of Africa during the Holocene Green Sahara Period. However, the current dry Sahara period has fragmented the WA rock dove populations as observed in the EEMS and *F*_ST_ results (Figure 2B; Figure S3). Further studies are necessary to confirm the geographic distribution of WA rock doves and analyze their genetics to validate these conclusions.

### 4.2 WA Rock Doves' Speciation Model

A question remains: why, despite their success in expanding westward, did the Sahara rock doves not invade and replace the WA rock doves? We believe WA rock doves might occupy a distinct ecological niche, related to the more tropical, forested, and flatter regions such as the Sahel shrublands and savannas, which prevented the Sahara rock dove's expansion. Urban et al. (1986) offer a hint, noting that Mali's WA rock dove populations exhibit partially arboreal behavior. Unfortunately, little is known about the ecology, behavior, and precise distribution of WA rock doves (van Grouw and Hernández‐Alonso 2025). Considering the genetic and likely ecological differentiation, we support the recognition of the WA rock dove as a distinct species, *C. gymnocycla*.

Based on the currently accessible data, we propose the following hypothetical scenario to explain the WA rock dove's speciation process: First, divergence could have increased while the two rock dove lineages were allopatric during the LGM, possibly due to local adaptation, genetic drift, and/or sexual selection (Butlin 1987). Once the lineages expanded again after the LGM, a hybrid zone would have been established in West Africa. The hybrid zone was maintained either as a tension zone defined by selection against the hybrids, a geographical selection gradient with selection against genotypes not locally adapted, or both (Wielstra 2021). Hybrid zones are often established in environmental gradients or near physical barriers that reduce population densities (Barton and Hewitt 1985). Consequently, introgression could have occurred by backcrossing of hybrids predominantly with WA rock doves. Introgression is known to be common in hybrid zones (Wielstra 2021), and barriers to gene flow can be asymmetric, favoring introgression in one direction (Barton and Hewitt 1985). Finally, natural selection may have promoted homogametic mating, increasing assortative mating and sexual selection, thereby promoting speciation by reinforcement (Butlin 1987).

In the context of local adaptation, random mating may decrease due to recombination disrupting locally adapted gene complexes, and migration may be reduced due to lower fitness in distinct environments (Lenormand 2012). The possible higher specialization of WA rock doves may explain their limited expansion, contrasting with the wide distribution observed in Central Asian lineage populations. Additionally, it has been observed that highly developed orbital rings with distinct colorations have appeared independently in several Columbiform species as ornaments. This may be due to sexual selection processes (Baptista et al. 2009), as could be the case with WA rock doves. Strong sexual selection produces high levels of assortative mating, and these both increase the probability of reinforcement (Butlin 1987).

### 4.3 Study Limitations

While our results offer new insights into the evolutionary history of rock doves, some limitations could be addressed with additional data to further enhance the depth and resolution of our findings. For example, future ecological and behavioral research on WA rock doves is essential to directly test the hypotheses generated in this study. Additionally, the inclusion of high‐quality genomes from modern specimens across each studied population would enable testing of key hypotheses related to selection signals associated with local adaptation, demographic changes over time, and estimates of admixture timing.

### 4.4 Taxonomic Implications

Currently, WA rock doves are recognized as *C. l. gymnocycla* Grey, 1856 (corrected from the original *C. l. gymnocyclus* by David and Gosselin 2002). This taxon is distributed in limited and isolated areas in Western Africa. It is differentiated from other populations mainly by a darker colouration than the nominate *C. l. livia*, and the presence of an extended, bright scarlet orbital ring (Gibbs et al. 2001; Baptista et al. 2009). Previous genomic analyses concluded that the WA rock doves should be considered a full species due to their high genetic divergence and be basal to all other rock doves (Hernández‐Alonso et al. 2023). Our analyses agree with the previously proposed taxonomic rearrangement, indicating high divergence of the WA rock doves possibly associated with a process of speciation by reinforcement. Following the Evolutionary species concept (Wiley 1978), we consider the evidence gathered sufficient to warrant the recognition of WA rock doves as a separate species, *C. gymnocycla* Grey, 1856.

Regarding the intraspecific classification of *C. livia* (excluding WA rock doves), Hernández‐Alonso et al. (2023) suggested that the species could be considered monotypic. This conclusion was based on the detection of 3 subspecies with apparent feral origin, the low genetic differentiation among populations, and their contiguous distribution that displays clinal morphological variation. The inclusion of new Sahara rock dove genomes in our analyses indicated that Sahara rock doves, *C. l. targia*, form the sister clade of ECA rock doves and domestic pigeons, are strongly isolated from other populations and present moderate to high genetic differentiation. On the other hand, the rest of the rock dove populations showed low to moderate genetic differentiation (Figure S3). Furthermore, the Red Sea populations, which obtained slightly higher *F*_ST_ values when compared to other *livia* rock doves, showed higher than average migration rates when compared to other coastal African populations (Figure 2B). For these reasons, we believe our genetic analyses indicate that *C. livia* might be formed by at least two subspecies. These are the nominate *C. l. livia* Gmelin, 1789 (including Eurasian and African populations from the Mediterranean and the Red Sea, and excluding the subspecies of feral origin: *atlantis*, *canariensis* and *dakhlae*), and the Sahara rock doves *C. l. targia* Geyr von Schweppenburg, 1916. In order to validate this rearrangement, morphological traits should be analyzed, as well as the genetics of other rock dove populations poorly explored so far.

### 4.5 Conservation Implications

Fragmented and isolated populations face higher probabilities of extinction because of inbreeding, loss of genetic diversity, and the accumulation of deleterious mutations (Frankham et al. 2019). In addition, habitat loss caused by climate change, urbanization, and environmental deterioration increases pressure on these populations, accelerating their collapse (Schlaepfer et al. 2018). As shown by our results, WA rock dove populations are fragmented, with apparently limited or no gene flow among them. They also exhibit very high inbreeding coefficients, similar to values reported for endangered species (Reed et al. 2002). For these reasons, and considering that WA rock doves represent a differentiated taxon, it may be relevant to reevaluate their conservation status to ensure their protection.

Similarly, our results indicate that the Sahara rock doves exhibit high inbreeding and significant isolation. However, their populations seem to have higher gene flow and greater heterozygosity. Notably, the mountainous regions of the Sahara remain far from human influence today, reducing the negative effects of anthropogenic activities on their populations.

Importantly, we did not find signals of admixture between domestic pigeons and either the WA or Sahara rock doves in our historical samples' dataset (Figures S2 and S7). However, admixture may have occurred and increased in more recent times, particularly among WA rock dove populations located closer to regions with significant rural and urban development that promote the presence of feral birds (Johnston and Janiga 1995). Studying modern populations will therefore be crucial to assess the extent and recency of admixture and its impact on the WA rock dove's genetic integrity. Finally, the Sahara rock doves, likely isolated from feral pigeons, may represent one of the last wild rock dove populations preserving their original genetic composition, free from the introgression of domestic genes. For this reason, Sahara rock doves are of high relevance for the conservation of *C. livia* rock doves. Currently, the species is categorized as “Least Concern” by the IUCN Red List of Threatened Species (2022), despite recurring expert concerns about potential genetic replacement due to continuous admixture with feral pigeons and habitat loss (Johnston et al. 1988; Johnston 1992; Johnston and Janiga 1995; Baptista et al. 1997).

## Author Contributions

**Germán Hernández‐Alonso:** conceptualization (lead), formal analysis (lead), funding acquisition (lead), visualization (lead), writing – original draft (lead). **Hein van Grouw:** resources (lead), writing – review and editing (equal). **Motahare F. Farahani:** resources (lead), writing – review and editing (equal). **Torsten Günther:** supervision (lead), writing – review and editing (equal).

## Disclosure

The results presented here have a direct impact on shaping conservation strategies to preserve important differentiated rock dove populations. Additionally, by making our data and results accessible through public databases, we contribute to the advancement of future scientific research and collaborative projects, fostering broader benefits for biodiversity conservation and evolutionary studies.

## Conflicts of Interest

The authors declare no conflicts of interest.

## Supporting information


**Data S1:** ece372061‐sup‐0001‐Supinfo.pdf.

## Data Availability

Generated raw sequence reads and the read alignment files mapped to the *C. livia* reference genome (Cliv_2.1) are deposited to the European Nucleotide Archive (Project ID: PRJEB74699). The generated pseudo‐haploid dataset is available in the Figshare repository: 10.6084/m9.figshare.29267132. The code used to perform the analyses is available in the Figshare repository: 10.6084/m9.figshare.29655956.
