# Supplementary material for: Gone With the Wind: Exploring a Vanished Rock Dove, Columba livia, Hybrid Zone in the Sahara Desert
Source: Ecol Evol. 2025 Aug 27;15(9):e72061. doi: 10.1002/ece3.72061 (PMC12391023; doi:10.1002/ece3.72061)
Supplement: Supplementary file 1 — Data S1: ece372061‐sup‐0001‐Supinfo.pdf. [file ECE3-15-e72061-s001.pdf]

Supplemental Information for:

Gone with the Wind: exploring a vanished rock dove, *Columba livia*, hybrid zone in the Sahara Desert

Germán Hernández-Alonso, Hein van Grouw, Motahare F. Farahani, Torsten Günther

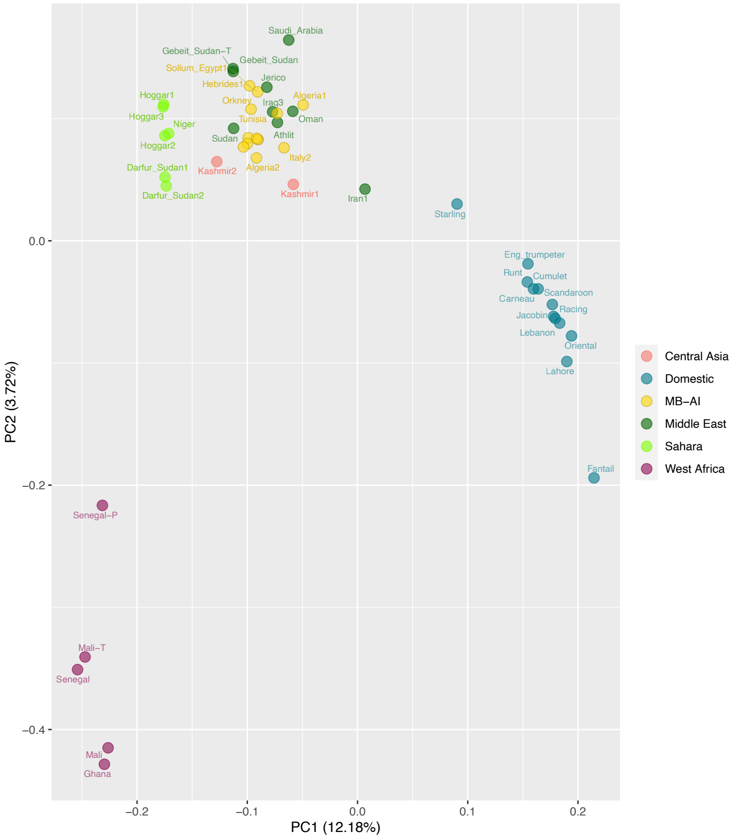

S1. Principal component analysis (PCA) based on genotype likelihoods. All samples were included for the analysis except for the outgroups. The observed population structure shows three main cluster: domestic pigeons, Eurasian-coastal African (ECA) rock doves, and West African rock doves. The later, appear as divergent compared to the other clusters, separated by PCA1 and PC2. Domestic pigeons are mainly separated from the ECA rock doves by PC 2. Finally, Sahara rock doves are slightly separated from ECA rock doves by the PC1. The PC1 represents 12.18% of the total variation in the analysis, while the PC2 represents 3.72% of the total variation. Colours represent different geographic regions, with MD-AI standing for Mediterranean Basin and Atlantic Islands.

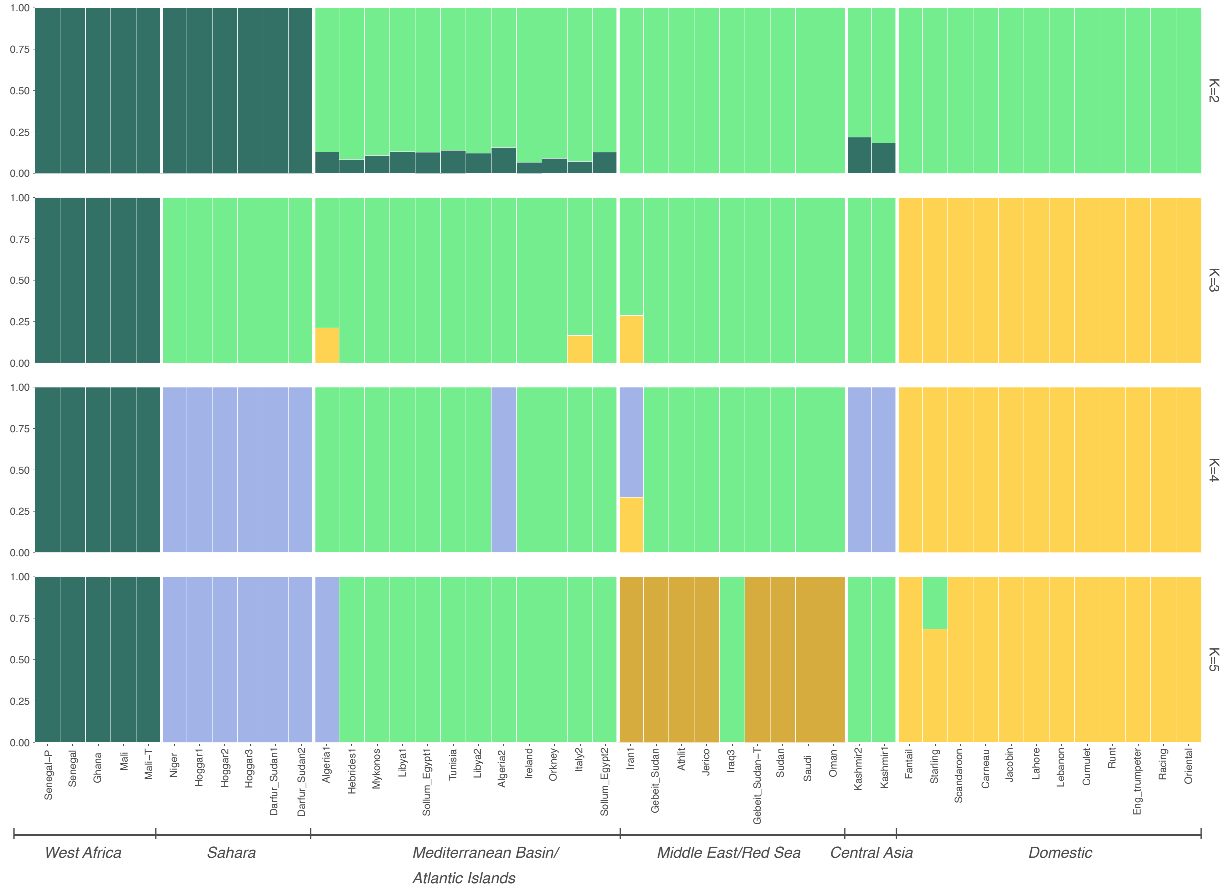

S2. Clusterisation analysis implemented in ADMIXTURE assuming 2-5 ancestry components (K). Each horizontal bar represents a rock dove or domestic pigeon genome. Different colors indicate the estimated ancestry components and their proportions. WA and Sahara rock doves do not show presence of other ancestry components.

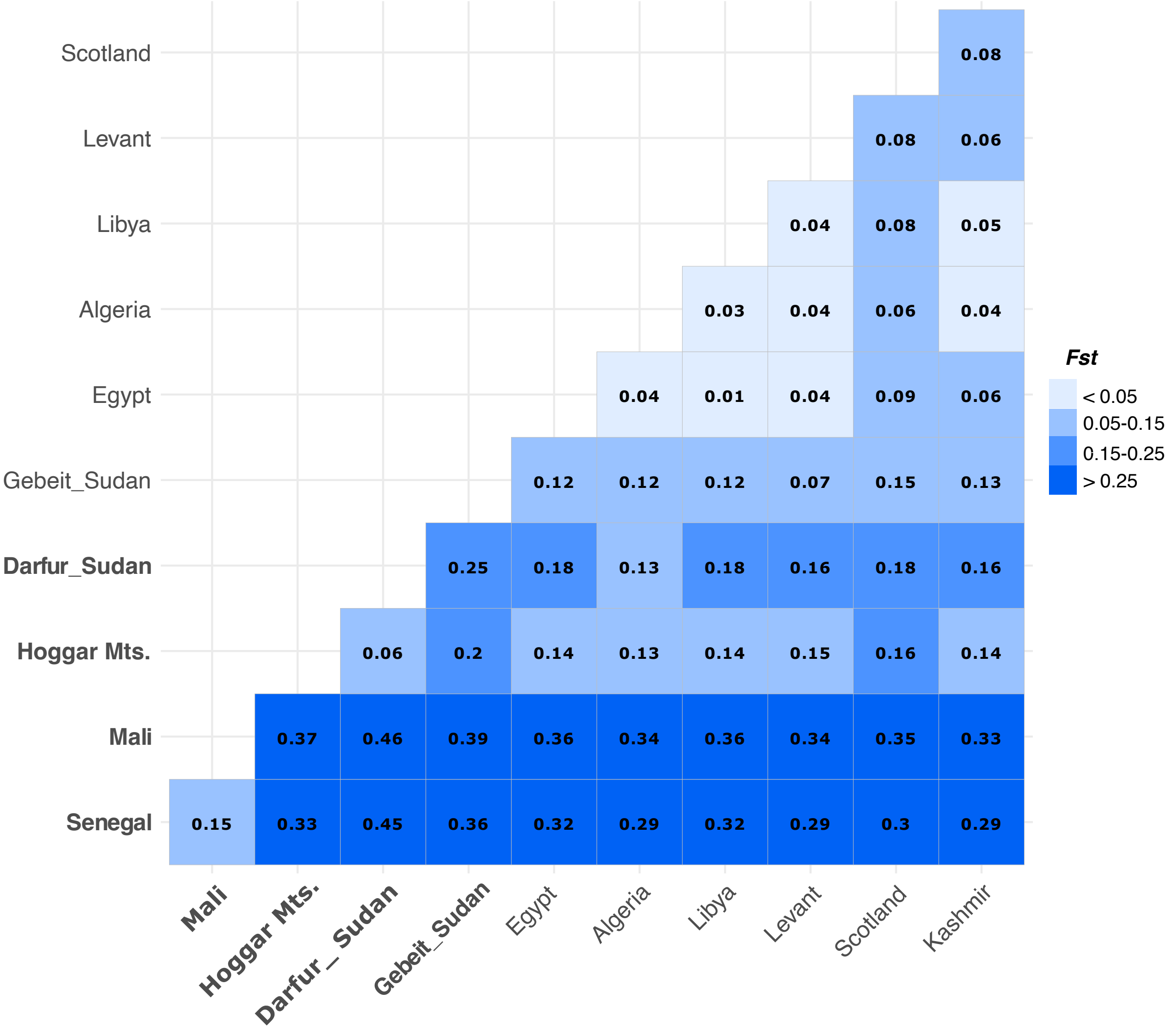

S3. Weighted pairwise fixation index (*Fst*). For a correct comparison, 2 genomes per same geographic region were selected to define the population. Values are interpreted as follows: 0–0.05 little genetic differentiation; 0.05-0.15 moderate differentiation; 0.15-0.25 high differentiation; over 0.25 very high genetic differentiation.

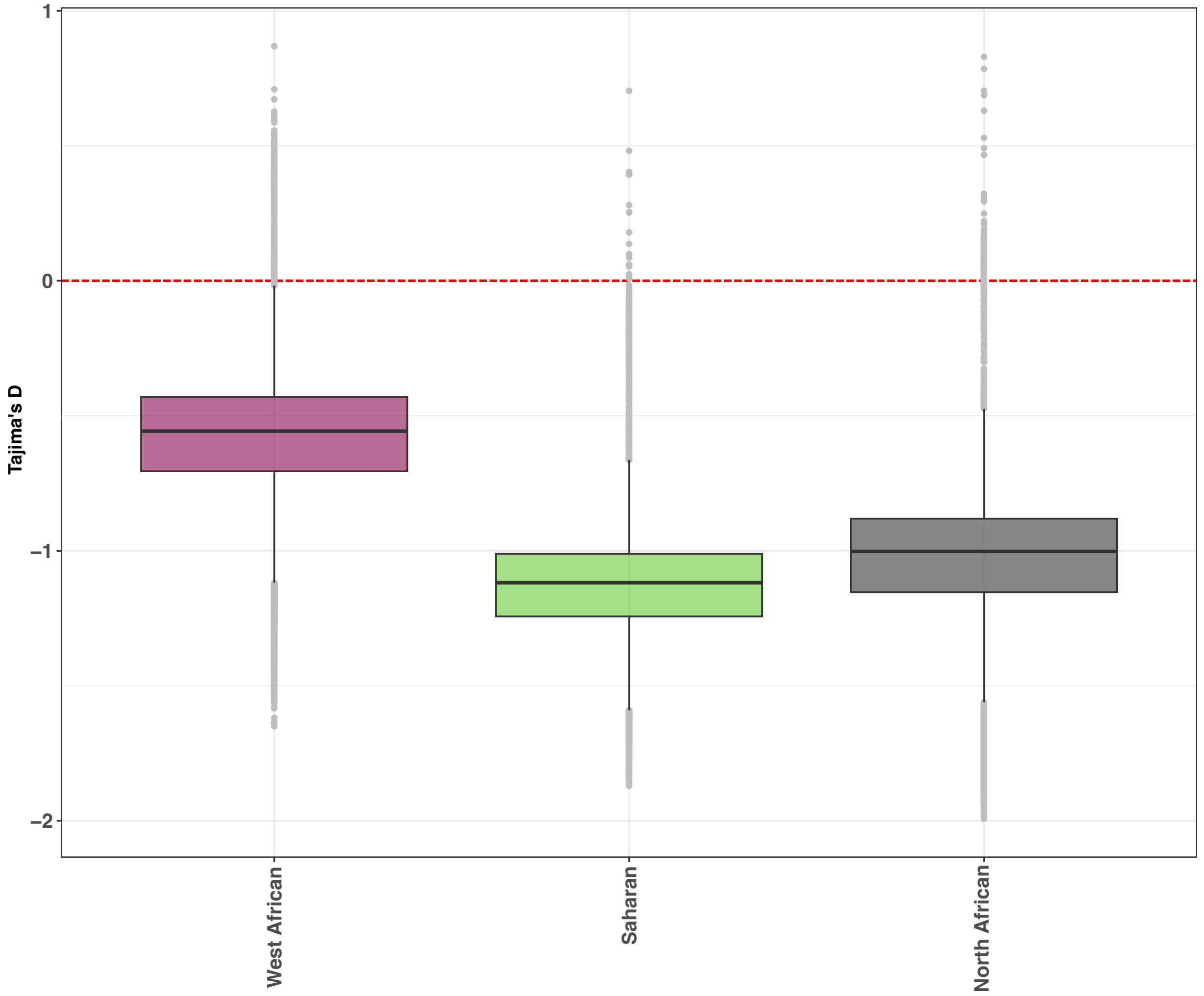

S4. Per-window Tajima's D estimated for three rock dove populations from Africa. The populations were defined in as follows: West African (Mali, Mali-T, Senegal, Senegal-P, Ghana), Sahara (Hoggar1, Hoggar2, Hoggar3, Niger, Darfur_Sudan1, Darfur_Sudan2), and North African (Sollum_Egypt1, Sollum_Egypt2, Libya1, Libya2, Algeria1, Algeria2, Tunes). The obtained Tajima's D values are predominantly negative indicating an excess of rare variants.

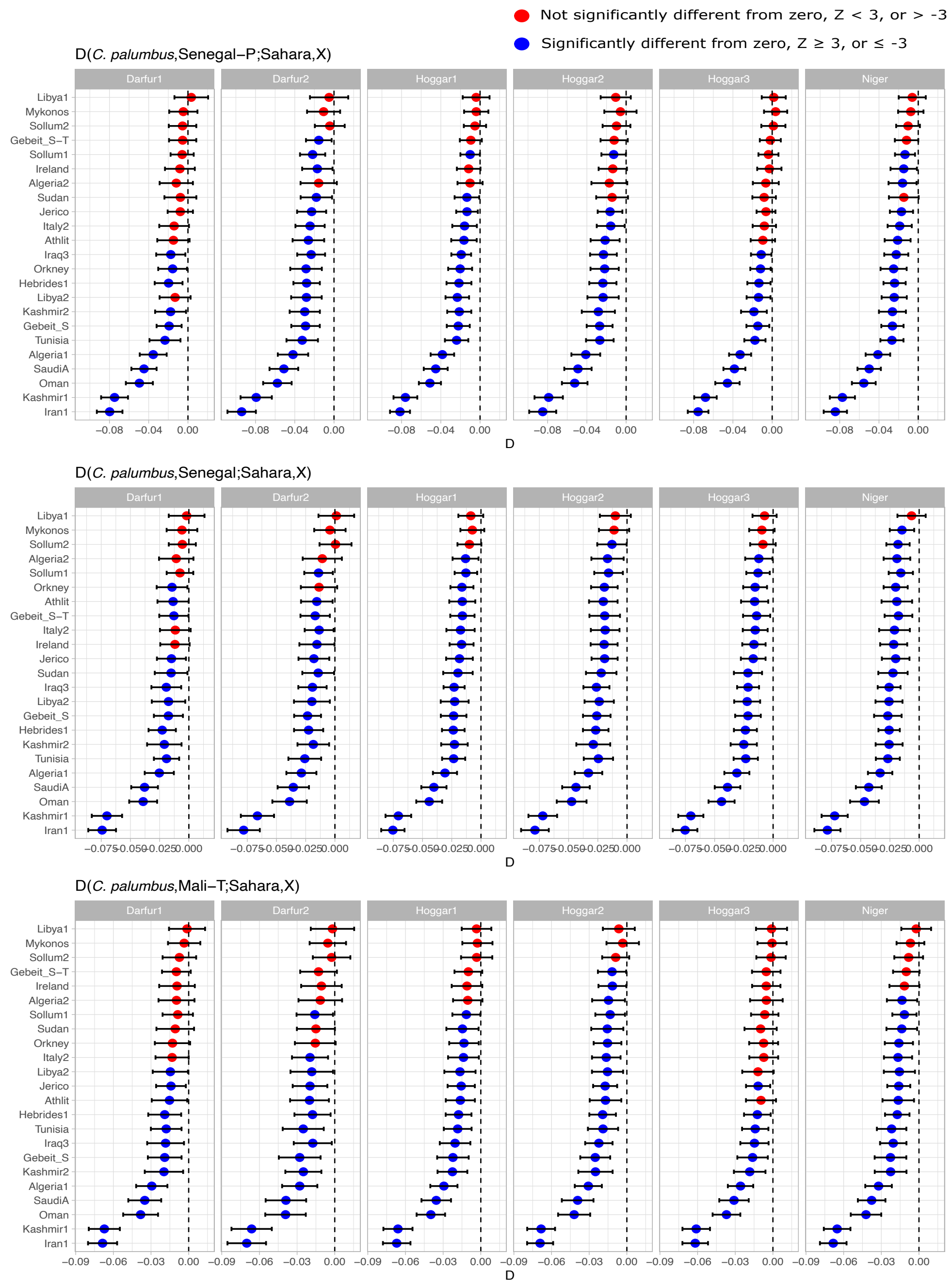

S5. *D*-statistics analyses testing admixture between each WA genome and each Sahara rock dove genome. The form of the test is shown at the top of each set of plots. All results present a tendency toward negative values, and most of them are statistically significative indicating admixture between the WA and Sahara rock doves.

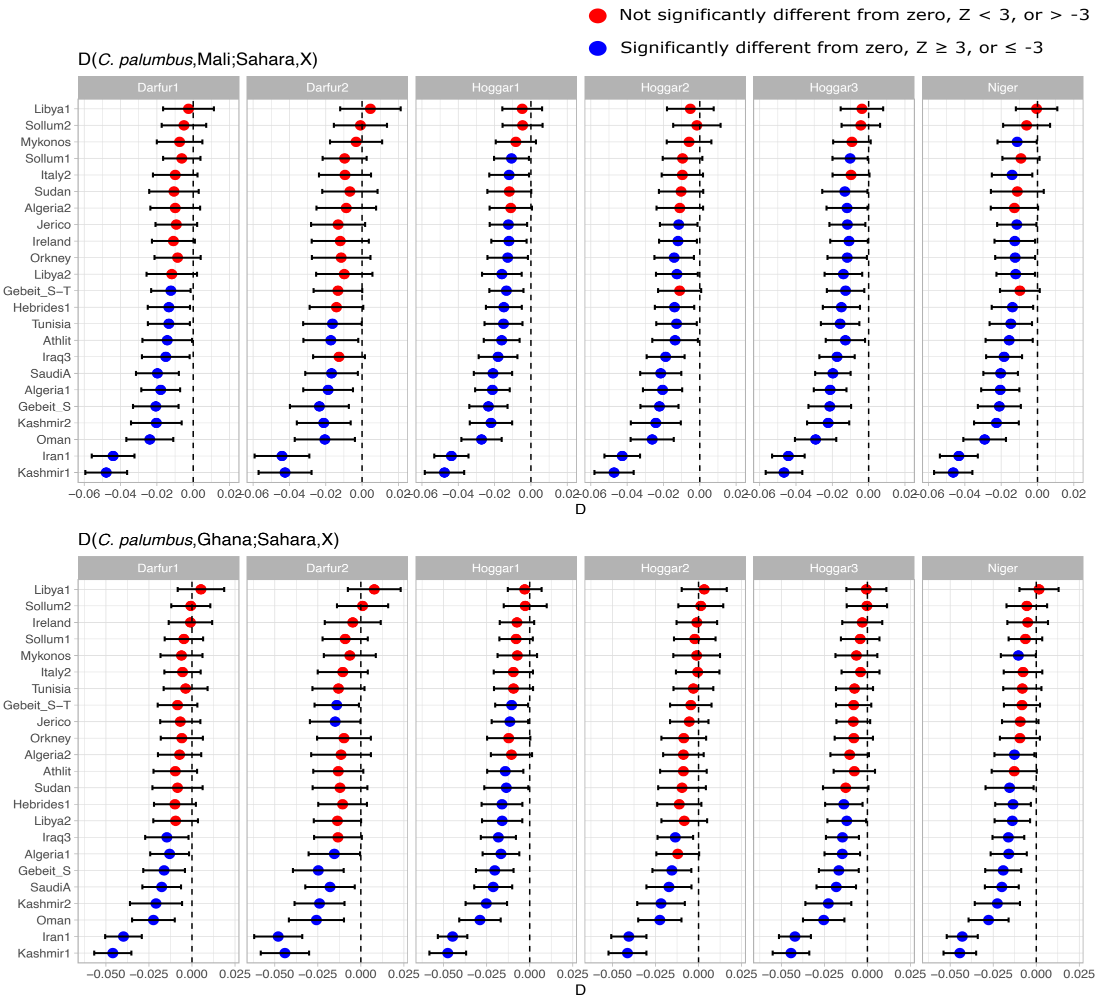

S6. Continuation of figure S4 showing *D*-statistics analyses testing admixture between each WA genome and each Sahara rock dove genome.

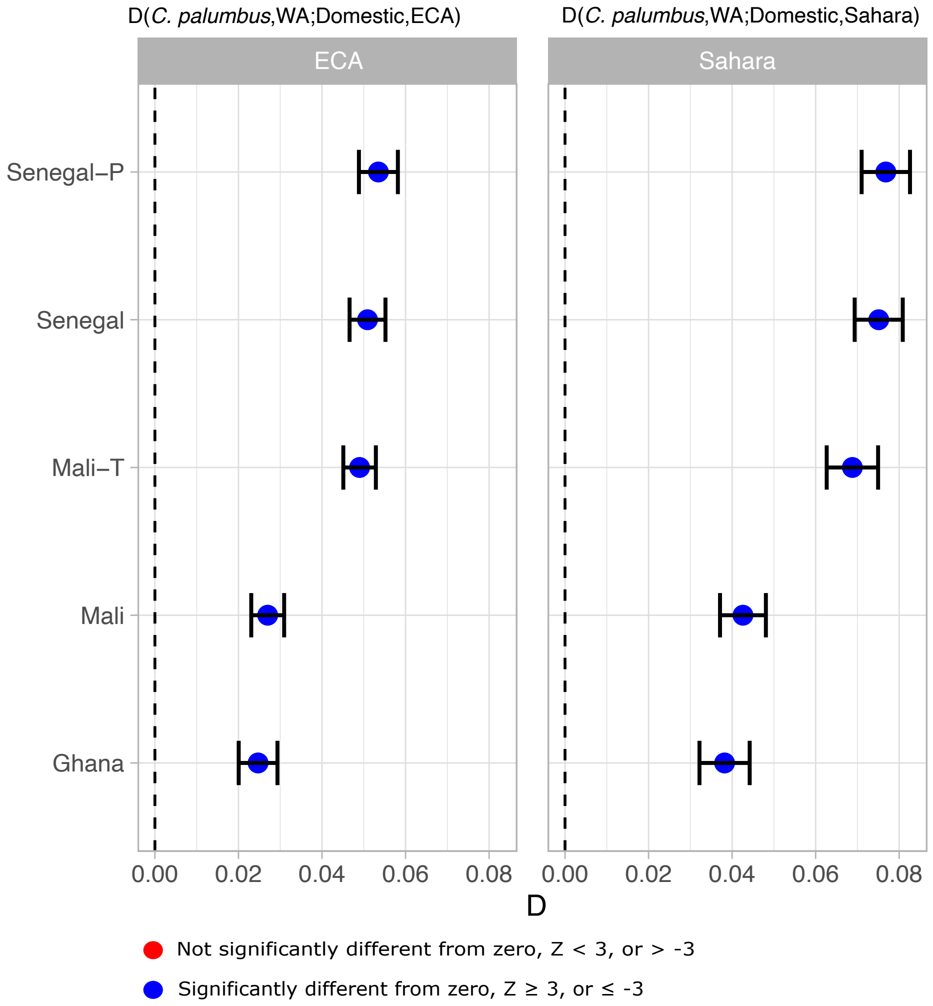

S7. *D*-statistics analyses testing admixture between WA rock doves and domestic pigeons. The tests were performed in the form *D*(*C. palumbus*, WA; domestic pigeons, ECA rock doves) and *D*(*C. palumbus*, WA; domestic pigeons, Sahara rock doves). The obtained *D* values are positive and statistically significant, indicating admixture signals between WA and wild rock doves. The *D* values are always higher in tests where the Sahara rock doves are included.

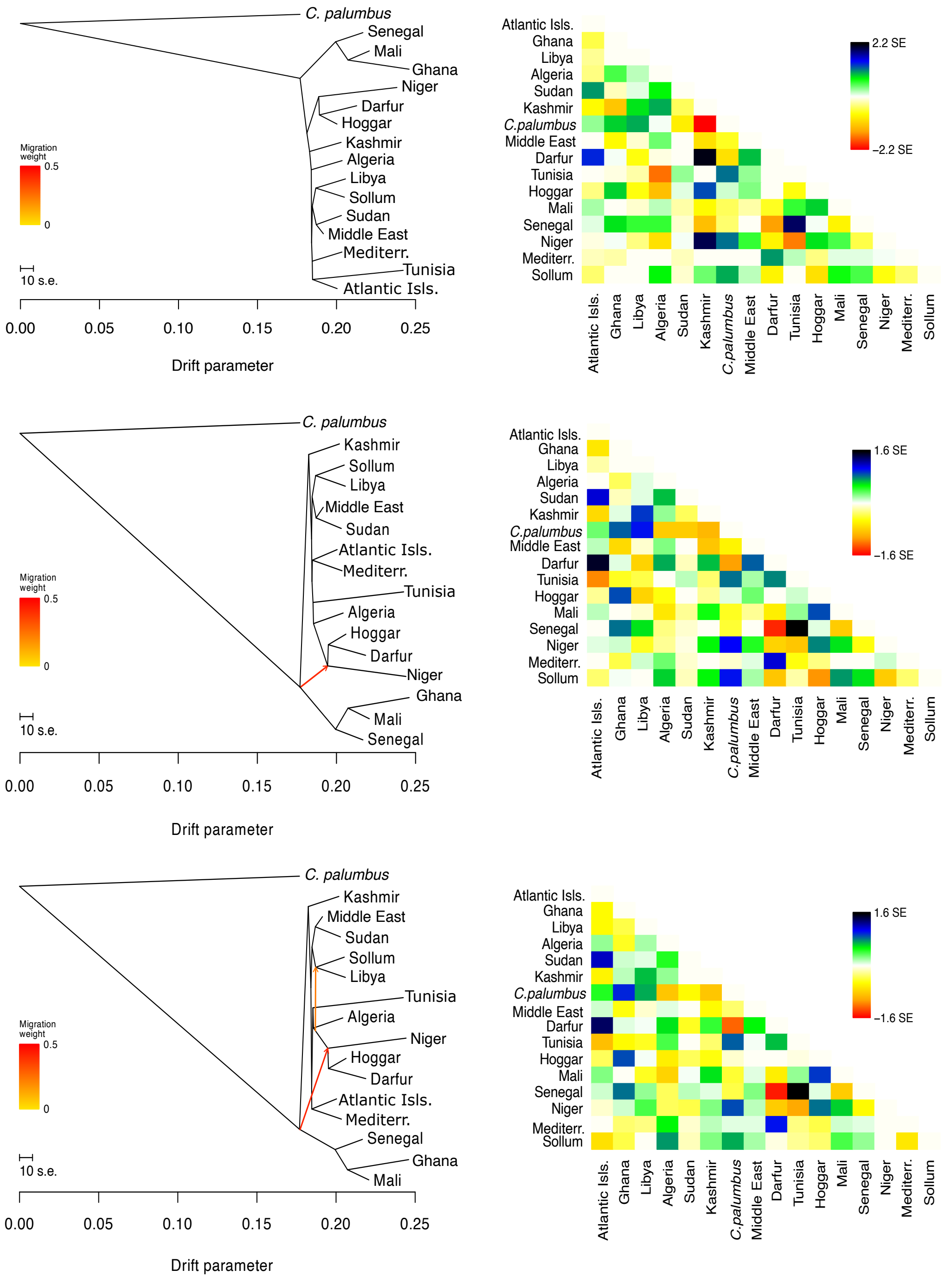

S8. OrientAGraph admixture graphs and residual plots estimated for 0 to 2 migration events (m). When OrientAGraph was used to estimate 1 m, an admixture event between the WA rock dove clade and the Sahara rock dove's clade is detected. The result for m=2 identified a new admixture event between the Sahara rock dove's branch and the coastal Africa clade formed by the populations from Sollum and Libya.

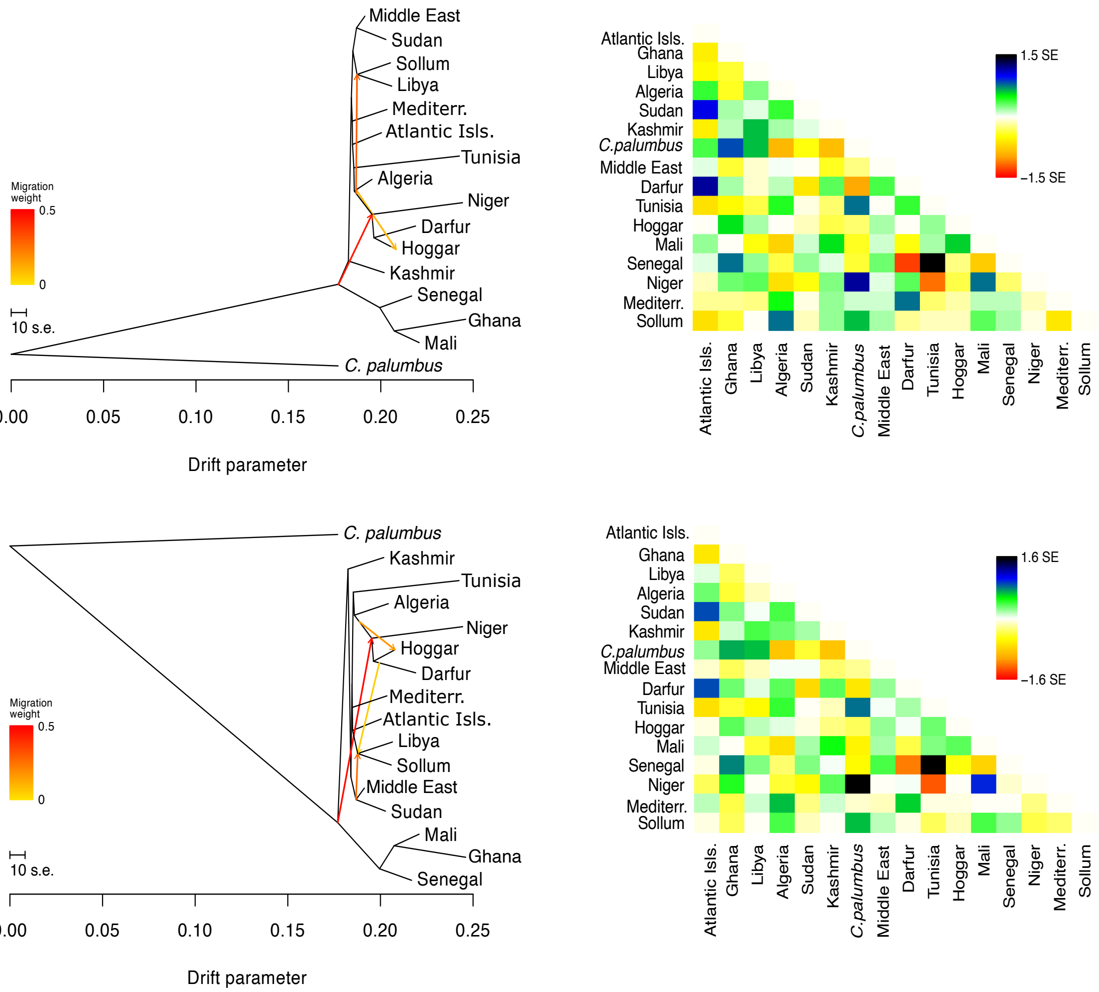

S9. OrientAGraph admixture graphs and residual plots estimated for 3 and 4 migration events (m). A third admixture event was detected between the Sahara rock doves from Hoggar and the population from coastal Algeria (m=3). Lastly, when m=4 was estimated, an additional admixture event was detected between the Sahara rock doves from Darfur and the Sollum-Libya clade.

Table S1. Metadata, including collection information and description of the processed genomic data.

**Samples**	**MuseimID**	**Common name**	**Species**	**Subspecies**	**Tissue**	**Sex (museum)**	**Date**	**LAB ID**	**Total number of pairs**	**retained reads**	**hits_length**	**% Endogenous**	**% PCR duplicates**	**Coverage (Cliv2.1)**	**MT depth of coverage**	**Zchr coverage (colLiv2)**	**Avg. Autosomal Coverage (colLiv2)**	**Sex**	**DNA damage (%) at 3' G>A**	**DNA damage (%) at 5' C>T**
P1 (Niger)	BMNH 1965.M.4617	Rock dove	*Columba livia*	*targia*	Dried toepad	male	1920	A194-001-L1P1	135310794	127263057	48.25	68.6	43.9	2.13	44.3889	1.8	2	Male	2.8	3.2
P2 (Hoggar1)	BMNH 1965.M.4625	Rock dove	*Columba livia*	*targia*	Dried toepad	male	1931	A194-002-L1P1	208948341	202953627	51.16	69.5	47.6	3.41	96.819	3.25	3.45	Male	2	2
P3 (Senegal-P)	BMNH 1855.12.19.299	Rock dove	*Columba livia*	*gymnocyclus*	Dried toepad	unknown	19th Century	A194-003-L1P1	182618633	175835508	41.05	66	66.9	1.42	70.8574	1.37	1.41	Male	3	3.3
P4 (Hoggar2)	BMNH 1965.M.4619	Rock dove	*Columba livia*	*targia*	Dried toepad	female	1931	A194-004-L1P1	161076879	153610506	50.11	68.8	56.8	2.06	75.6897	1.3	2.17	Female	1.9	2.4
P5 (Hoggar3)	BMNH 1965.M.4618	Rock dove	*Columba livia*	*targia*	Dried toepad	female	1931	A194-005-L1P1	162323838	157912605	61.38	69.1	46	3.26	96.0822	1.88	3.32	Female	2	2.2
P6 (Senegal)	BMNH 1920.6.8.151	Rock dove	*Columba livia*	*gymnocyclus*	Dried toepad	female	1920	A194-006-L1P1	184338185	178205876	51.12	68.1	44.9	3.08	85.874	1.74	3.11	Female	2.1	2

Table S2. Whole samples dataset used in this study.

Project ID	Species	subspecies	Location	Coverage	Sample	Project/BioSample	Publication/Source
P1 (Niger)	*Columba livia*	*targia*	Niger	1.88	P1	BioSample: SAMEA115481299	This study
P2 (Hoggar1)	*Columba livia*	*targia*	Algeria	3.27	P2	BioSample: SAMEA115481300	This study
P3 (Senegal-P)	*Columba livia*	*gymnocyclus*	Senegal	1.42	P3	BioSample: SAMEA115481301	This study
P4 (Hoggar2)	*Columba livia*	*targia*	Algeria	2.02	P4	BioSample: SAMEA115481302	This study
P5 (Hoggar3)	*Columba livia*	*targia*	Algeria	3.07	P5	BioSample: SAMEA115481303	This study
P6 (Senegal)	*Columba livia*	*gymnocyclus*	Senegal	2.87	P6	BioSample: SAMEA115481304	This study
Algeria1	*Columba livia*	*livia*	Algeria	6.90442	mRD02	BioSample: SAMEA113570299; Accession: ERS15565291	Hernández-Alonso *et al*. 2023
Algeria2	*Columba livia*	*livia*	Algeria	1.32118	mRD27	BioSample: SAMEA113570324; Accession: ERS15565316	Hernández-Alonso *et al*. 2023
Tunisia	*Columba livia*	*gaddi*	Tunisia	3.73506	mRD22	BioSample: SAMEA113570319; Accession: ERS15565311	Hernández-Alonso *et al*. 2023
Libya1	*Columba livia*	/	Libya	1.45356	mRD09	BioSample: SAMEA113570306; Accession: ERS15565298	Hernández-Alonso *et al*. 2023
Libya2	*Columba livia*	*gaddi*	Libya	1.64367	mRD23	BioSample: SAMEA113570320; Accession: ERS15565312	Hernández-Alonso *et al*. 2023
Sollum_Egypt1	*Columba livia*	/	Egypt	3.63235	mRD10	BioSample: SAMEA113570307; Accession: ERS15565299	Hernández-Alonso *et al*. 2023
Sollum_Egypt2	*Columba livia*	/	Egypt	1.30504	mRD62	BioSample: SAMEA113570359; Accession: ERS15565351	Hernández-Alonso *et al*. 2023
Ghana	*Columba livia*	*gymnocycla*	Ghana	2.66716	mRD37	BioSample: SAMEA113570334; Accession: ERS15565326	Hernández-Alonso *et al*. 2023
Mali	*Columba livia*	*gymnocycla*	Mali	2.40762	mRD38	BioSample: SAMEA113570335; Accession: ERS15565327	Hernández-Alonso *et al*. 2023
Mali-T	*Columba livia*	*lividor*	Mali	2.25644	mRD56	BioSample: SAMEA113570353; Accession: ERS15565345	Hernández-Alonso *et al*. 2023
Gebeit_Sudan-T	*Columba livia*	*butleri*	Sudan	2.95078	mRD53	BioSample: SAMEA113570350; Accession: ERS15565342	Hernández-Alonso *et al*. 2023
Gebeit_Sudan	*Columba livia*	*schimperi*	Sudan	2.84193	mRD14	BioSample: SAMEA113570311; Accession: ERS15565303	Hernández-Alonso *et al*. 2023
Sudan	*Columba livia*	/	Sudan	1.28812	mRD54	BioSample: SAMEA113570351; Accession: ERS15565343	Hernández-Alonso *et al*. 2023
Darfur_Sudan1	*Columba livia*	*targia*	Sudan	1.11694	mRD55	BioSample: SAMEA113570352; Accession: ERS15565344	Hernández-Alonso *et al*. 2023
Darfur_Sudan2	*Columba livia*	*targia*	Sudan	0.815965	mRD59	BioSample: SAMEA113570356; Accession: ERS15565348	Hernández-Alonso *et al*. 2023
Oman	*Columba livia*	/	Oman	2.0479	mRD65	BioSample: SAMEA113570362; Accession: ERS15565354	Hernández-Alonso *et al*. 2023
Saudi Arabia	*Columba livia*	*palaestinae*	Saudi Arabia	8.17008	mRD61	BioSample: SAMEA113570358; Accession: ERS15565350	Hernández-Alonso *et al*. 2023
Hebrides1	*Columba livia*	*livia*	Scotland	3.7448	mRD06	BioSample: SAMEA113570303; Accession: ERS15565295	Hernández-Alonso *et al*. 2023
Orkney	*Columba livia*	*livia*	Scotland	2.74103	mRD32	BioSample: SAMEA113570329; Accession: ERS15565321	Hernández-Alonso *et al*. 2023
Ireland	*Columba livia*	*livia*	Ireland	1.71761	mRD30	BioSample: SAMEA113570327; Accession: ERS15565319	Hernández-Alonso *et al*. 2023

Iran1	*Columba livia*	*gaddi*	Iran	5.24834	mRD01	BioSample: SAMEA113570298; Accession: ERS15565290	Hernández-Alonso *et al*. 2023
Iraq3	*Columba livia*	*gaddi*	Iraq	3.43691	mRD20	BioSample: SAMEA113570317; Accession: ERS15565309	Hernández-Alonso *et al*. 2023
Jerico	*Columba livia*	*gaddi*	Palestine	3.14747	mRD19	BioSample: SAMEA113570316; Accession: ERS15565308	Hernández-Alonso *et al*. 2023
Athlit	*Columba livia*	*gaddi*	Palestine	2.15011	mRD16	BioSample: SAMEA113570313; Accession: ERS15565305	Hernández-Alonso *et al*. 2023
Italy2	*Columba livia*	*livia*	Italy	2.07794	mRD34	BioSample: SAMEA113570331; Accession: ERS15565323	Hernández-Alonso *et al*. 2023
Mykonos	*Columba livia*	*livia*	Greece	1.84985	mRD07	BioSample: SAMEA113570304; Accession: ERS15565296	Hernández-Alonso *et al*. 2023
Kashmir1	*Columba livia*	*neglecta*	Pakistan	2.64795	mRD39	BioSample: SAMEA113570336; Accession: ERS15565328	Hernández-Alonso *et al*. 2023
Kashmir2	*Columba livia*	*neglecta*	Pakistan	1.6238	mRD05	BioSample: SAMEA113570302; Accession: ERS15565294	Hernández-Alonso *et al*. 2023
Starling	*Columba livia*	*domestica*	USA	9.53322316	BGI_PI-AF	BioSample: SAMN01057555; Sample name: BGI_PI-AF; SRA: SRS346887	Shapiro *et al*. 2013
Runt	*Columba livia*	*domestica*	USA	13.6122688	BGI_PI-BH	BioSample: SAMN01057562; Sample name: BGI_PI-BH; SRA: SRS346894	Shapiro *et al*. 2013
Carneau	*Columba livia*	*domestica*	USA	10.5936475	BGI_PI-AQ	BioSample: SAMN01057550; Sample name: BGI_PI-AQ; SRA: SRS346882	Shapiro *et al*. 2013
Fantail	*Columba livia*	*domestica*	USA	23.0139271	BGI_fan	BioSample: SAMN01057533; Sample name: BGI_fan; SRA: SRS346865	Shapiro *et al*. 2013
Lahore	*Columba livia*	*domestica*	USA	11.9221745	BGI_PI-AU	BioSample: SAMN01057565; Sample name: BGI_PI-AU; SRA: SRS346897	Shapiro *et al*. 2013
Cumulet	*Columba livia*	*domestica*	USA	9.90656583	BGI_PI-AZ	BioSample: SAMN01057570; Sample name: BGI_PI-AZ; SRA: SRS346902	Shapiro *et al*. 2013
Jacobin	*Columba livia*	*domestica*	USA	8.58698763	BGI_PI-AT	BioSample: SAMN01057553; Sample name: BGI_PI-AT; SRA: SRS346885	Shapiro *et al*. 2013
Scandaroon	*Columba livia*	*domestica*	USA	9.64042197	BGI_PI-AH	BioSample: SAMN01057541; Sample name: BGI_PI-AH; SRA: SRS346873	Shapiro *et al*. 2013
Racing	*Columba livia*	*domestica*	USA	9.89515096	BGI_PI-DI	BioSample: SAMN01057535; Sample name: BGI_PI-DI; SRA: SRS346867	Shapiro *et al*. 2013
Oriental	*Columba livia*	*domestica*	USA	6.77444122	BGI_PI-DJ	BioSample: SAMN01057536; Sample name: BGI_PI-DJ; SRA: SRS346868	Shapiro *et al*. 2013
Lebanon	*Columba livia*	*domestica*	USA	10.9323751	BGI_PI-AV	BioSample: SAMN01057566; Sample name: BGI_PI-AV; SRA: SRS346898	Shapiro *et al*. 2013
English trumpeter	*Columba livia*	*domestica*	USA	12.7604383	BGI_PI-BJ	BioSample: SAMN01057564; Sample name: BGI_PI-BJ; SRA: SRS346896	Shapiro *et al*. 2013
C. rupestris	*Columba rupestris*	/	USA	14.7358478	BGI_C-rup	BioSample: SAMN01057534; Sample name: BGI_C-rup; SRA: SRS346866	Shapiro *et al*. 2013
C. palumbus	*Columba palombus*	/	USA	20.1297516	Copal	BioSample: SAMN04886480; Sample name: Copal; SRA: SRS1416881	Vickrey et al. 2018
C. larvata	*Columba larvata*	/	/	3.52264353	Aplar	BioSample: SAMN04886481; Sample name: Aplar; SRA: SRS1416882	Vickrey et al. 2018
C. guinea	*Columba guinea*	/	/	21.8830195	Cogui	BioSample: SAMN04886479; Sample name: Cogui; SRA: SRS1416880	Vickrey et al. 2018
P. fasciata	*Patagioneas fasciata*	/	USA	3.74713643	BTP2013	BioSample: SAMN04386172; Sample name: BTP2013; SRA: SRS2103361	Murray et al. 2017
